# Evolution of Genes Involved in Gamete Interaction: Evidence for Positive Selection, Duplications and Losses in Vertebrates

**DOI:** 10.1371/journal.pone.0044548

**Published:** 2012-09-05

**Authors:** Camille Meslin, Sylvie Mugnier, Isabelle Callebaut, Michel Laurin, Géraldine Pascal, Anne Poupon, Ghylène Goudet, Philippe Monget

**Affiliations:** 1 UMR85 Physiologie de la Reproduction et des Comportements, INRA, Nouzilly, France; 2 UMR6175, CNRS, Nouzilly, France; 3 Université François Rabelais de Tours, Tours, France; 4 IFCE, Nouzilly, France; 5 Département Agronomie Agro-équipement Élevage Environnement, AgroSup Dijon, Dijon, France; 6 IMPMC UMR 7590, Université Pierre et Marie Curie-Paris 6, Paris, France; 7 UMR 7207, CNRS/MNHN/UPMC, Muséum National d’Histoire Naturelle, Paris, France; Université Paris-Sud, France

## Abstract

Genes encoding proteins involved in sperm-egg interaction and fertilization exhibit a particularly fast evolution and may participate in prezygotic species isolation [1], [2]. Some of them (ZP3, ADAM1, ADAM2, ACR and CD9) have individually been shown to evolve under positive selection [3], [4], suggesting a role of positive Darwinian selection on sperm-egg interaction. However, the genes involved in this biological function have not been systematically and exhaustively studied with an evolutionary perspective, in particular across vertebrates with internal and external fertilization. Here we show that 33 genes among the 69 that have been experimentally shown to be involved in fertilization in at least one taxon in vertebrates are under positive selection. Moreover, we identified 17 pseudogenes and 39 genes that have at least one duplicate in one species. For 15 genes, we found neither positive selection, nor gene copies or pseudogenes. Genes of teleosts, especially genes involved in sperm-oolemma fusion, appear to be more frequently under positive selection than genes of birds and eutherians. In contrast, pseudogenization, gene loss and gene gain are more frequent in eutherians. Thus, each of the 19 studied vertebrate species exhibits a unique signature characterized by gene gain and loss, as well as position of amino acids under positive selection. Reflecting these clade-specific signatures, teleosts and eutherian mammals are recovered as clades in a parsimony analysis. Interestingly the same analysis places *Xenopus* apart from teleosts, with which it shares the primitive external fertilization, and locates it along with amniotes (which share internal fertilization), suggesting that external or internal environmental conditions of germ cell interaction may not be the unique factors that drive the evolution of fertilization genes. Our work should improve our understanding of the fertilization process and on the establishment of reproductive barriers, for example by offering new leads for experiments on genes identified as positively selected.

## Introduction

A lot of barriers are able to prevent a successful reproduction. Dobzhansky proposed a classification of species isolation from prezygotic to postzygotic isolation [5]. In postzygotic isolation, a zygote is formed but the hybrid offspring is not viable or sterile. In prezygotic isolation, species could not mate because of a difference in sexual behavior or for mechanical issues. Another step of prezygotic isolation is the lack of interaction between male and female gametes, the sperm-egg interaction being a several steps process that leads to a successful fertilization. Although details vary between species, a successful fertilization requires three main steps in sperm-egg interaction [6]. The first step is the recognition and the binding between sperm and the extracellular matrix surrounding eggs (ECM), called the chorion in teleosts, the vitelline envelope in birds, and the zona pellucida (ZP) in mammals. The second step, required for the sperm to penetrate through the egg coat, is the acrosome reaction. The third step is the binding and the subsequent fusion between sperm and the oolemma, the plasma membrane of the oocyte. Many experiments have been performed in order to determine the importance of candidate proteins during fertilization. Surprisingly, knock-out experiment of B4GT1 or targeted mutation that abolished the protease activity of ACR in mouse show that these proteins are important but not essential [7], [8]. Several studies showed that genes involved in sperm-egg interaction exhibit a particular evolution. For example, a recent study shows that several ZP genes have been lost during evolution of vertebrates [9]. ZP1 gene, one of the glycoproteins that compose the ZP, has been lost during evolution in cattle, pig, cat and dog, whereas ZP4 gene has been lost in mouse. Moreover, ZPAX and ZPD genes, which are present in chicken, have been lost in mammals. In addition, several genes involved in fertilization such as ZP3, ADAM1, ADAM2, ACR and CD9 have been shown to evolve under positive selection [3], [4]. The authors hypothesized that these positively selected amino acids might play a role in the specificity of sperm-egg interaction between species because they are located in the putative sperm-egg binding domain. Despite the fact that several studies suggested the involvement of positive Darwinian selection on sperm-egg interaction genes, none has been done exhaustively. In our study, we have listed all genes which have been experimentally shown to be involved in one vertebrate and in at least one step of the sperm-egg interaction, on both sperm and/or oocyte, and on 19 species with fully-sequenced genomes. We have studied all these genes with an evolutionary perspective by searching for gene gains and losses, as well as positive selection, a divergent evolution of these genes among vertebrate species potentially leading to reproductive isolation.

## Results and Discussion

### Rates of Gene Gain, Loss and Pseudogenization are Clade-dependent Variables

**Figure 1:**
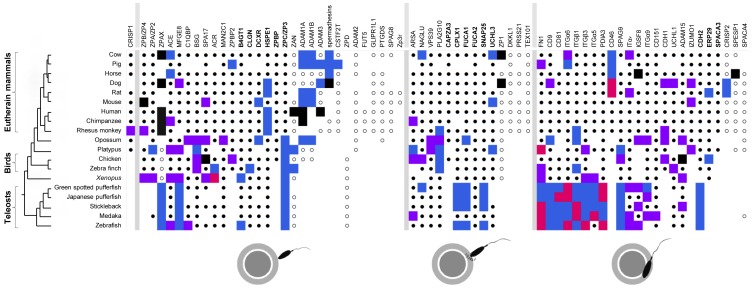
Signature of evolution of genes involved in sperm-oocyte interaction in Vertebrates. Genes (names are indicated on the top) are classified into three main functional groups (from left to right): sperm-zona pellucida binding, acrosome reaction, and sperm-oolemma fusion. Species are indicated in the tree (left). Blue square: duplication; purple square: positive selection; pink square: duplication + positive selection. Black square: pseudogene; Black dot: no event. Black circle: no calculation of positive selection. White square: no gene found.

**Figure 2:**
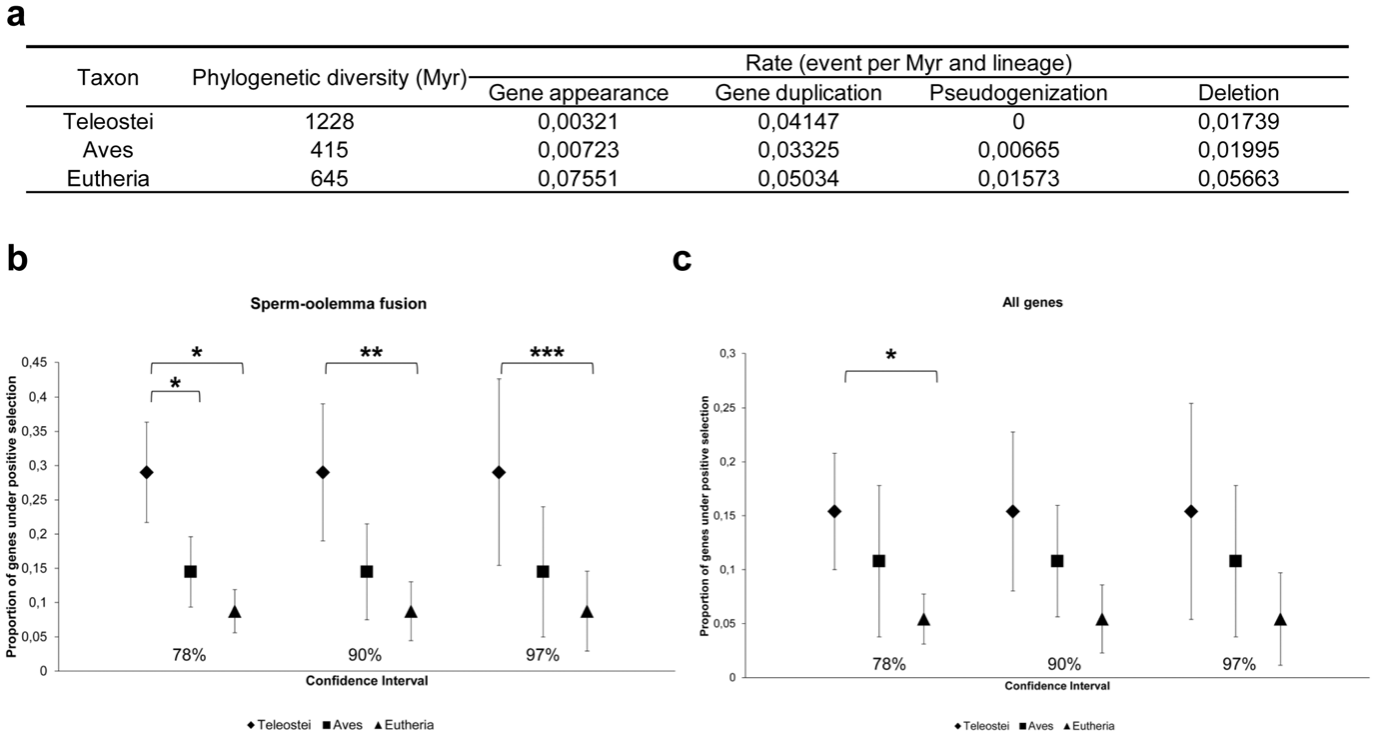
Rate of gene appearance, gene duplication, pseudogenization, loss and intensity of positive selection in Teleostei, Aves and Eutheria. a. Rate of events per million years (Myr) per lineage for the set of genes studied. This includes up to 69 genes, but in most cases, the number of relevant genes is lower because some types of events cannot happen in some taxa (losses or duplications cannot happen if a gene is primitively absent, and gains cannot occur in a clade if the gene appeared before the base of a given clade), and we have incorporated that factor into our calculations to obtain comparable rates across taxa. The geological age of lineages is used in the rate calculation through the phylogenetic diversity of the clades. **b** and **c.** Proportion of genes under positive selection in Teleostei, Aves and Eutheria for genes belonging to the third step, the sperm-oolemma fusion (B), and for all genes (C). Three types of confidence intervals are shown: 78%, 90% and 97% representing testing at 0.05, 0.01 and 0.001 probability thresholds, respectively. Statistically significant comparisons, for which confidence intervals do not overlap, are indicated.

We identified 69 genes from the literature (Figure 1 and Table S1) that encode proteins experimentally known to be involved in at least one of the three steps described above. These genes are well studied in mouse and cattle that are common models for studies on physiology and reproduction. We classified all genes into three main groups according to which step they are involved in, excepted CRISP1 that is involved in all three steps of fertilization [10], [11], [12]. Thanks to phylogenetic analyses, we found several gene duplications, as well as pseudogenizations, among genes involved in the three main steps of fertilization. In particular, we found evidence for (i) gain of FUT5 and Zp3r genes in primates and rodents, respectively, (ii) pseudogenization of SPA17 and ADAM15 genes in chicken, ZAN, ADAM1a and ADAM3 in human, ADAM3 in chimpanzee, spermadhesins in dog, IGSF8 and SPESP1 in horse, as well as ZPAX in dog, and the known ZP pseudogenes published elsewhere [9] (ZPB/ZP4 in mouse, ZP1 in cow and dog, ZPAX in primates and cow); and (iii) specific duplication events of ADAM genes in therian mammals, spermadhesins in ungulates, as well as the ACR gene in *Xenopus* (14 copies) and HSPE1 in macaque (11 copies). For each gene, we have systematically assessed, by optimizing gene presence, number, and type (functional or pseudogene) on a time-calibrated reference phylogeny, the absolute rate of the following four types of events: gene gain, duplication, inactivation (resulting in a pseudogene) and complete loss (presumably through deletion, although incomplete genome annotation may have led to overestimation of loss rate). The overall rate of gene appearance (genes that appear only in some monophyletic groups), pseudogenization and deletion events differs strongly between clades and type of event (Figure 2a). By taking into consideration the number and the geological age of sampled lineages, we found that these three types of events are far more frequent in eutherians than in birds and teleosts. A phylogeny-informed binomial test shows that these differences between eutherians and teleosts are statistically significant, differences between eutherians and birds are also statistically significant for deletions and gene appearance. However, a possible bias stems from the fact that our list of gene is composed almost exclusively of genes identified in eutherians and thus, the rates of the different events may be overestimated in this clade. No pseudogene has been identified in teleosts. The duplications occur at more comparable rates across these three taxa but tend to occur slightly more frequently in eutherians than in teleosts and aves. The consensual conclusion is that duplication rates for most of the genes are higher in teleosts than in other taxa due to the third whole genome duplication, specific to teleosts [13], [14]. Consequently, our results are surprising. They reflect the greater geological age of lineages of teleosts, resulting in a phylogenetic diversity twice as great as eutherians, despite a lower number of included species (Figure 2a). Thus, a greater number of events per lineage is required in teleosts to yield comparable rates.

### Genes of Teleosts are More Subjected to Positive Selection

We have also submitted the protein sequence to the PhyleasProg web server [15] (http://phyleasprog.inra.fr/) to determine selective pressures that shape the evolution of these genes and used a new phylogenetically-informed test to compare the amount of positive selection in various clades. The calculation of positive selection is globally based on rate of non-synonymous (*dN*) and synonymous (*dS*) substitutions [16]. The accumulation of synonymous substitutions (saturation of *dS*) may create a problem to detect positive selection with sequences from highly divergent species such as human and zebrafish with some methods. However, a previous study showed that the method we use to detect positive selection, i.e. maximum likelihood estimate, is robust to *dS* saturation [17]. The calculation was possible for 48 genes and we found amino acids under positive selection for 33 of them (Figure 1). Among teleosts, birds and eutherians taxa, we found various patterns of positive selection. The FN1 gene has sites under positive selection in 7 species for which data are available. The number of genes under positive selection in each species ranges from 0 (horse and human) to 10 (opossum). Positive selection occurs in the three groups of genes, suggesting the presence of an evolutionary pressure in the three steps of fertilization.

Our comparison of the intensity of positive selection shows that genes involved in sperm-oolemma fusion, including genes of the integrin family (IGSF8, ITGα9, ITα_v_, ITGα6), are mostly subjected to positive selection in teleosts, but much less so in mammals (Figure 2b). The difference between teleosts and birds (p<0.05) and between teleosts and eutherian mammals (p<0.001) are statistically significant whereas the difference between birds and eutherian mammals is not. This result is surprising for teleosts because we expected to find more amino acids under positive selection for genes involved in the sperm-ECM binding, which would contribute to the first barrier in free-spawning conditions. In particular, positive Darwinian selection has been shown to play a role in the rapid evolution of genes involved in sperm-egg binding in three other free-spawning taxa, sea urchin, mussel and abalone [18], [19], [20], [21]. The comparisons of the intensity of positive selection between taxa for sperm-ECM binding and between taxa for acrosome reaction are not significant. When analyzed globally, genes involved in the three steps of gamete interaction show a similar, but less marked, pattern in which the only statistically significant difference is between teleosts and eutherian mammals (p<0.05) (Figure 2c). Besides, in contrast to previous studies [4], we did not find positive selection for ZPC/ZP3, in agreement with more recent data obtained on a larger number of species [22]. We also show that several genes are positively selected in more than one species, such as ACE that exhibits positive selection in chimpanzee, opossum, *Xenopus*, and zebrafish.

### Amino Acids under Positive Selection are not Located in known Functional Domains of Proteins

**Figure 3:**
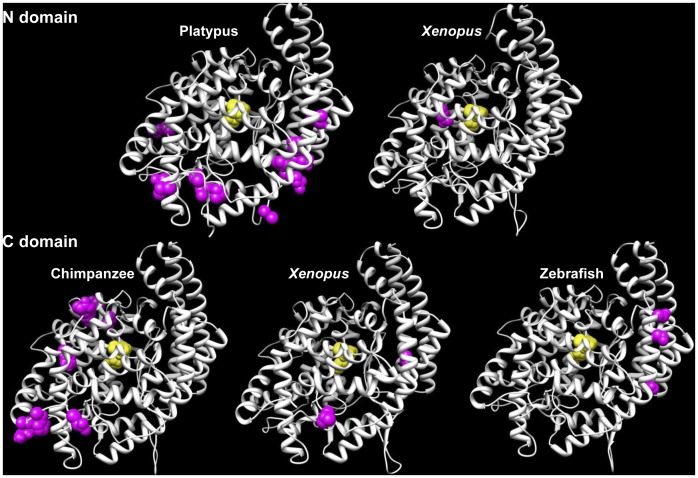
3D structures of N and C domain of ACE in platypus, *Xenopus*, chimpanzee and zebrafish. ACE structures have been modeled for four species based on human PDB structure (PDB: 1O8A) [36]. This enzyme is composed of two homolog N and C domains, each containing a catalytic site composed by two histidines in a conserved -HEXXH- zinc-binding motif [37] and a glutamate as the third ligand 24 residues downstream this motif [38]. These three binding sites are indicated in yellow, whereas amino acids under positive selection are in pink. ACE protein is expressed by sperm. Gene knockout experiments of ACE in mouse results in an impaired uterotubular sperm migration and a reduced ability to bind the zona pellucida of the egg [39], [40], [41].

In order to project our previous results of positive selection on 3D structures, we have built 23 tridimensional models of 6 proteins (ACE, ARSA, C1QBP, ITα_V_, PDIA3 and UCHL3) from species where positive selection has been detected (Figure 3 and Figure S1 to S5). These 6 proteins were chosen because PDB template structures were available [23]. Firstly, we show that amino acids under positive selection are never located in the domain involved in known biological role (for ACE, ARSA, PDIA3 and UCHL3). This suggests that these residues are involved in other secondary functions such as interaction with partners or in the folding of the protein. Secondly, we show that amino acids under positive selection, for the same protein, have different location between species. Thus, we suppose that the function of these proteins in the process of fertilization may differ between species. This hypothesis is supported by a recent study that has emphasized the importance of structural architecture of the egg coat and its importance in fertilization [24]. Thus, we postulate that positive Darwinian selection could contribute to slight modifications of the architecture of egg or sperm coat by changing key amino acids in some taxa. These architectural modifications may also create or reinforce reproductive barriers. Experimental investigations must be done (i) to identify amino acids involved in sperm-egg interaction in these proteins, (ii) to study the expression and the role of these genes in gametes and (iii) to identify their function in sperm-egg interaction in a greater number of species.

In conclusion, we study the evolution of 69 genes involved in the sperm-egg interaction. This work should help to improve our understanding of the evolution and the mechanism of fertilization in vertebrates, which still remains puzzling. In particular, the evolution of proteins present on male and female gametes can lead to reproductive barriers at the level of sperm-egg interaction in vertebrates. The variation between species regarding positive selection patterns as well as gain or loss of certain genes involved in fertilization could actually lead to the appearance or the reinforcement of reproductive barriers. The relationship between positive selection and molecular adaptation has been experimentally shown previously [25], [26]. Our work provides a list of potential targets, i.e. amino acids under positive selection, for targeted mutagenesis experiments to see if these amino acids are indeed involved in reproductive barriers between species. However, this list of genes should be completed after further experiments on other species. All this should improve significantly our understanding of the appearance of prezygotic isolation mechanisms, and hence, of the speciation process, which is of paramount importance in generating biodiversity.

## Materials and Methods

### Selection of Data

This study has sampled the genome of 19 bony vertebrate species that have been fully sequenced (*Bos taurus*, *Sus scrofa*, *Equus caballus*, *Canis familiaris*, *Rattus norvegicus*, *Mus musculus*, *Homo sapiens*, *Pan troglodytes*, *Macaca mulatta*, *Monodelphis domestica*, *Ornithorhynchus anatinus*, *Gallus gallus*, *Taeniopygia guttata*, *Xenopus tropicalis*, *Tetraodon nigroviridis*, *Takifugu rubripes*, *Gasterosteus aculeatus*, *Oryzias latipes* and *Danio rerio*). For all identified genes, the corresponding Ensembl protein ID was retrieved from Ensembl database and submitted to the PhyleasProg web server [15]. For each submitted Ensembl protein ID, the PhyleasProg web server interrogates the Ensembl database to retrieve orthologs and paralogs, the orthology/paralogy relationships assignment being based on a phylogenetic approach in Ensembl. All phylogenetic trees were reconstruct by PhyleasProg using the TreeBeST program (http://treesoft.sourceforge.net/treebest.shtml) and carefully examined before interpreting selective pressure results, also calculated by PhyleasProg. Selective pressure results are systematically verified and eventually corrected by synteny analysis as previously described by Tian, et al. [27]. Thus, calculations were performed with supposed correct orthologs. To score the presence, number and type of genes in the data matrix, a search for pseudogenes was systematically performed in the concerned genomes for genes for which no ortholog has been identified in at least one of the species of interest. The pseudogene status was inferred for a gene in a genome only if we have found a stop codon or an indel in the sequence identified by the similarity search in the syntenic locus in comparison with the other species of interest. Moreover, a search for duplicates was realized by using phylogenetic trees available in Ensembl and accessible via the Ensembl ID used for PhyleasProg and summarized in the Table S1.

### Inference of Positive Selection

The inference of selection was performed by PhyleasProg with branch-site models of codeml of the PAML package, with each ortholog branch tested for positive selection. Multiple alignments were systematically and carefully examined to avoid all false positive results. In particular, amino acids predicted to be under positive selection that were at the boundary of the alignments were not considered because doubtful. We also eliminated genes for which positive selection was due to sequence errors in Ensembl according to a comparison with other available sequences from other database such as RefSeq in NCBI (Figure S6 to S8). Each branch of each phylogenetic tree was tested for positive selection. So we performed multiple test corrections by controlling for the false discovery rate (FDR) using the R package QVALUE [28]. Results are considered significant with a threshold of *q* = 10% of false positives. Sites with posterior probabilities of Bayes Empirical Bayes analyses superior to 95 or 99% were considered as positively selected. Datasets with less than 10 sequences, the minimum threshold required to obtain significant results, with excessively divergent sequences, or with sequences of genes for which annotations are not reliable were not retained for subsequent analysis (Figure 1). MODELLER [29] was used to build homology models of the 3D structure of proteins when possible and amino acids under positive selection and essential functional residues were localized on them.

### Comparison of Evolutionary Rates and the Intensity of Positive Selection Across Clades

Character history was inferred using parsimony optimization onto our rooted tree. In most cases, this yielded an unambiguous polarity. In a few cases, such as for the character ZPB/ZP4, the ancestral condition could not be unambiguously resolved using parsimony; it was then resolved using additional criteria. For instance, in that case, one of the two main clades, Teleostei, had the state "absent", whereas the other main clade, Tetrapoda, had the state "single copy". In such cases, we assumed that absence was primitive, and scored a gain for Tetrapoda. All cases of losses located on other parts of the tree (not on one of the two daughter-branches of the basalmost node) were inferred from parsimony optimizations whose polarity, in that case, is unproblematic. Similarly, when one of the two basalmost branches can be inferred to have had a single copy whereas the other could most parsimoniously have two or more, we inferred that the presence of a single copy was primitive, based on the principle that the most common condition is likely to be primitive. This situation was encountered in the character ZPAX, for which Tetrapoda have a single copy, whereas Teleostei can most parsimoniously be inferred to have had two or three. To assess evolutionary rates, we optimized the 69 discrete characters representing gene presence, number and type (functional gene or pseudogene) onto the reference tree (Figure S9) and scored the occurrence of events of four types (gene appearance, duplication, transformation into a pseudogene, or loss) in three clades (Teleostei, Aves, Eutheria). We considered the basal branch subtending each of these three taxa as being part of it, and this is consistent with the fact that 19 sampled taxa in this study do not include representatives of both basalmost branches of these clades (e.g. paleognaths are not among the two birds sampled). We then divided the number of events of each type in each clade by that clade’s sampled phylogenetic diversity index [30] (the sum of branch lengths, including of the basal branch). We then divided the resulting number by the proportion of the 69 genes in which such changes could occur in each clade. The last operation compensates for the fact that several genes present in eutherian mammals are absent in teleosts, and this avoids underestimating the rate of gene duplication in teleosts. Thus, origin of these genes could occur in teleosts, but not duplication, inactivation, or deletion. To statistically compare the rates between teleosts and eutherians, we took into consideration the sampled phylogenetic diversity of each clade and the number of genes for which each event was possible to assess the relative probability of occurrence of each event under the null hypothesis that these events are clade-independent. We then assessed the probability that a distribution at least as extreme as the observed one arises under the null hypothesis using a binomial test implemented in GraphPad (http://www.graphpad.com/quickcalcs/binomial2.cfm).

To compare the intensity of positive selection in the three clades of interest (Teleostei, Aves, Eutheria), we computed the inferred ancestral value of each clade using squared-change parsimony [31] in Mesquite [32]. Confidence intervals of these values were computed using phylogenetic independent contrasts [33] using the PDAP:PDTREE module [34] of Mesquite. These were used to assess statistical significance of the differences in ancestral values of each clade. Our method is derived from the continuous analysis developed for heterochrony analysis of continuous characters [35], a method whose statistical properties (especially power and type I error rate) have already been assessed using simulations. However, we improved a bit the method to more fully incorporate uncertainty about nodal estimates; instead of comparing a nodal value with the confidence interval (CI) of another clade, we compare the confidence intervals of both clades. This allows using narrower CIs because the probability that both nodal values actually lie outside the computed CI at a given error threshold is equal to the square of that threshold. As a result, instead of comparing a nodal value with the 95% CI (0.05 probability threshold), we compared the CIs at 78% of both clades because the square of 0.22 is just under 0.05 (we rounded off to the nearest higher percentage, which should make the test slightly conservative). Thus, if the two compared CIs do not overlap, we conclude that the difference is statistically significant. This method thus incorporates uncertainty in the estimates of all compared nodal values. The fact that data from all taxa are used to compute ancestral values should also make the test conservative because this should tend to make ancestral estimates of sister-taxa more similar to each other than if the ancestral values were estimated for each clade separately.

## Supporting Information

Figure S1 **3D structure of ARSA in chicken, chimpanzee and medaka.** The three structures were modeled with human ARSA structure as template (PDB: 1N2K) [105]. The active site of the enzyme is represented in yellow, amino acids under positive selection in pink. ARSA is expressed by the spermatozoa; its involvement in the fertilization process was demonstrated with antibodies directed against ARSA. When the sperm is pretreated with these antibodies, their ability to bind the zona pellucida is reduced [71].
(TIF)

Figure S2 **3D structures of C1QBP in opossum and zebrafish.** Both structures were modeled with human P32 as template (PDB: 1P32) [106]. Amino acids under positive selection are in pink. The C1QBP glycoprotein is localized on sperm; its participation in the fertilization process was demonstrated using anti-C1QBP antibodies. The interaction between sperm and zona pellucida is suppressed when the sperm is pretreated with these antibodies [107].
(TIF)

Figure S3 **3D structures of ITα_v_ in cow, rat, green spotted pufferfish, medaka and zebrafish.** Four structures were modeled with human α_v_β3 integrin as template (PDB: 1L5G) [108]. The structure presented for the cow is the template itself. Amino acids under positive selection are in pink. This integrin is expressed by both oocyte and sperm. The pretreatment of sperm with anti ITα_v_ antibodies significantly decreases the sperm-zona pellucida binding and the fertilization [109].
(TIF)

Figure S4 **3D structures of PDIA3 in green spotted pufferfish, japanese pufferfish and zebrafish.** The three structures were modeled with human ERp57 as template (PDB: 3F8U) [110]. Catalytic motifs of the protein are in yellow, amino acids under positive selection are in pink. PDIA3 is expressed on sperm in the acrosomal region. Its role in the fusion process of sperm and oolemma was demonstrated with the use of anti-PDIA3 antibodies [89].
(TIF)

Figure S5 **Models of the 3D structures of UCHL3 in opossum and stickleback.** The two structures were modeled with human UCHL3 as template (PDB: 1UCH) [111]. Amino acids corresponding to the catalytic site of the enzyme are represented in yellow, amino acids under positive selection in pink. UCHL3 protein is present on both sperm acrosome and oocyte cortex. A study showed that UCHL3 is an ubiquitin C-terminal hydrolase and is involved in antipolyspermy defense during porcine fertilization [78].
(TIF)

Figure S6 **An example of a sequence error in Ensembl.** The sequence presented in (**a)** is the protein sequence of the gene PDIA3 from *Xenopus*. Amino acids in red and orange are those which have been detected as positively selected by PhyleasProg. A tBLASTn has been performed with this sequence against EST database of NCBI. The result of the BLAST is presented in (**b)**, the domain which contains amino acids under positive selection in (**a)** is not retrieved. A new sequence for *Xenopus* was searched in NCBI RefSeq database. The sequence of the best BLAST hit is submitted to a tBLASTn against the EST database of NCBI for verification (**c**). The new identified sequence from NCBI replaced in this case the sequence from Ensembl.
(TIF)

Figure S7 **An example of a doubtful amino acid under positive selection.** The amino acid in red in the sequence of the rat (*Rattus norvegicus*) was found as positively selected in the alignment of protein sequences of the gene CD46. Because it is located at the boundary of the alignment, this amino acid is not considered, because doubtful.
(TIF)

Figure S8 **An example of reliable amino acids under positive selection.** Amino acids indicated in red in the sequence of the platypus (*Ornithorhynchus anatinus*) are considered as reliable in this multiple sequence alignment of ACE protein sequences.
(TIF)

Figure S9 **Time-calibrated reference phylogeny used for all phylogeny-informed analyses.** This tree follows classical time-calibrated phylogenies of teleosts [112] and mammals [113]. The divergence time between the chicken and zebra finch follows a recent review paper [114]. The other divergence times derive from a review of paleontological and molecular dates [115]. The names of several higher taxa are placed, with those most discussed in the text in bold type. The scale to the right gives approximate divergence times (in millions of years).
(TIF)

Table S1 **Genes included in the analysis.**
(DOCX)
