# Supplementary figures and images for: Evolution of Genes Involved in Gamete Interaction: Evidence for Positive Selection, Duplications and Losses in Vertebrates
Source: PLoS One. 2012 Sep 5;7(9):e44548. doi: 10.1371/journal.pone.0044548 (PMC3434135; doi:10.1371/journal.pone.0044548)

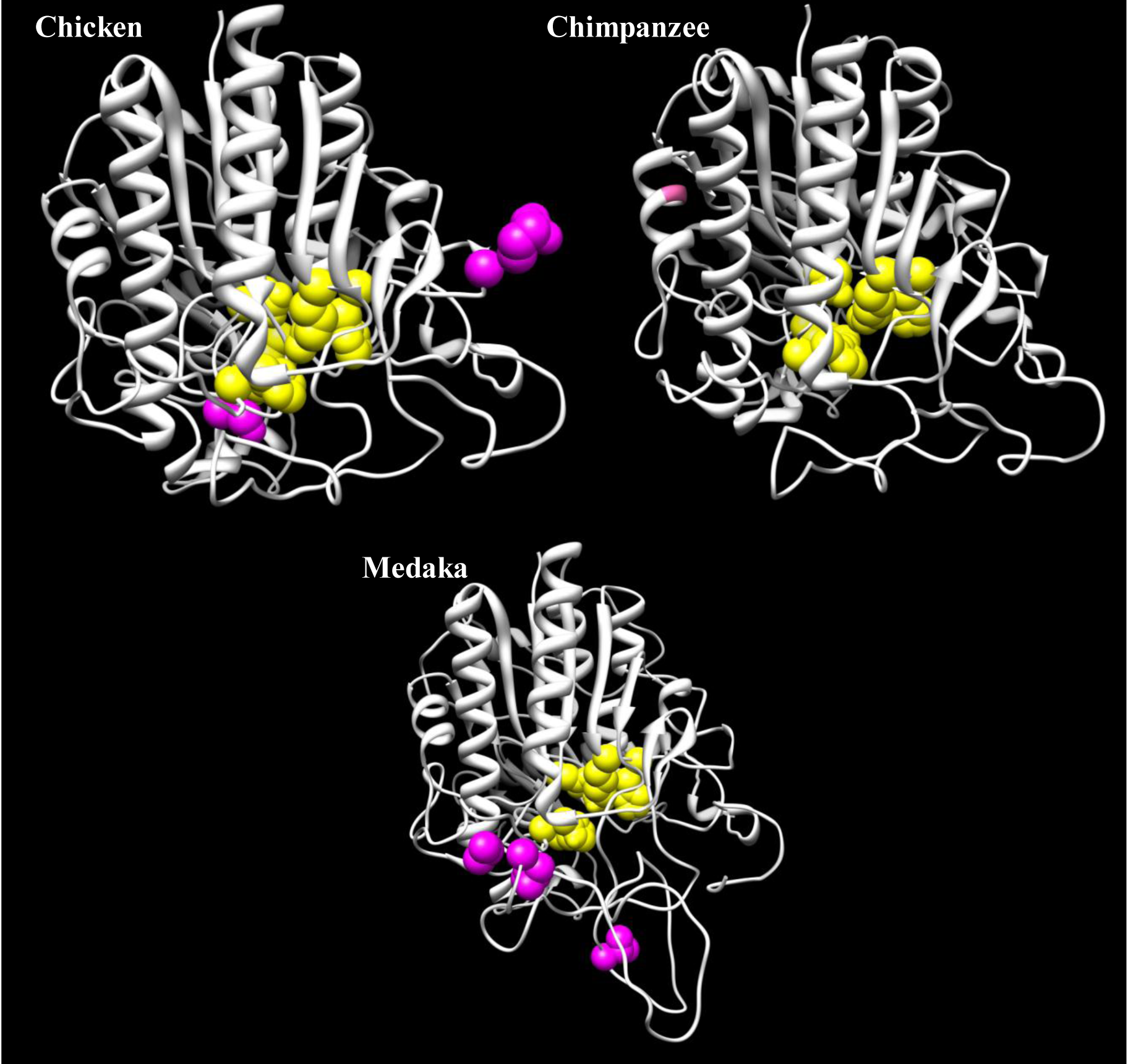

Supplement: Figure S1 — 3D structure of ARSA in chicken, chimpanzee and medaka. The three structures were modeled with human ARSA structure as template (PDB: 1N2K) [105]. The active site of the enzyme is represented in yellow, amino acids under positive selection in pink. ARSA is expressed by the spermatozoa; its involvement in the fertilization process was demonstrated with antibodies directed against ARSA. When the sperm is pretreated with these antibodies, their ability to bind the zona pellucida is reduced [71]. (TIF) [file pone.0044548.s001.tif]

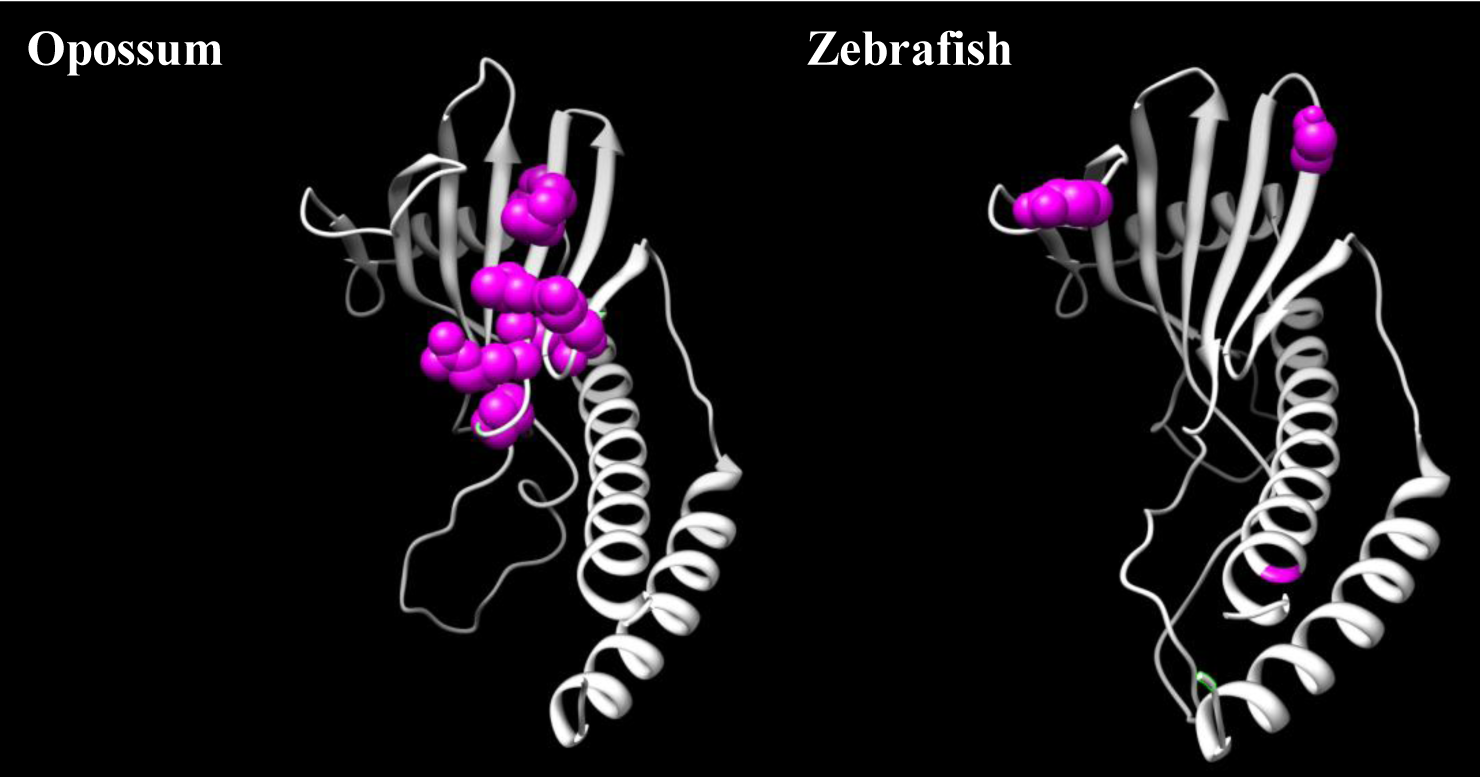

Supplement: Figure S2 — 3D structures of C1QBP in opossum and zebrafish. Both structures were modeled with human P32 as template (PDB: 1P32) [106]. Amino acids under positive selection are in pink. The C1QBP glycoprotein is localized on sperm; its participation in the fertilization process was demonstrated using anti-C1QBP antibodies. The interaction between sperm and zona pellucida is suppressed when the sperm is pretreated with these antibodies [107]. (TIF) [file pone.0044548.s002.tif]

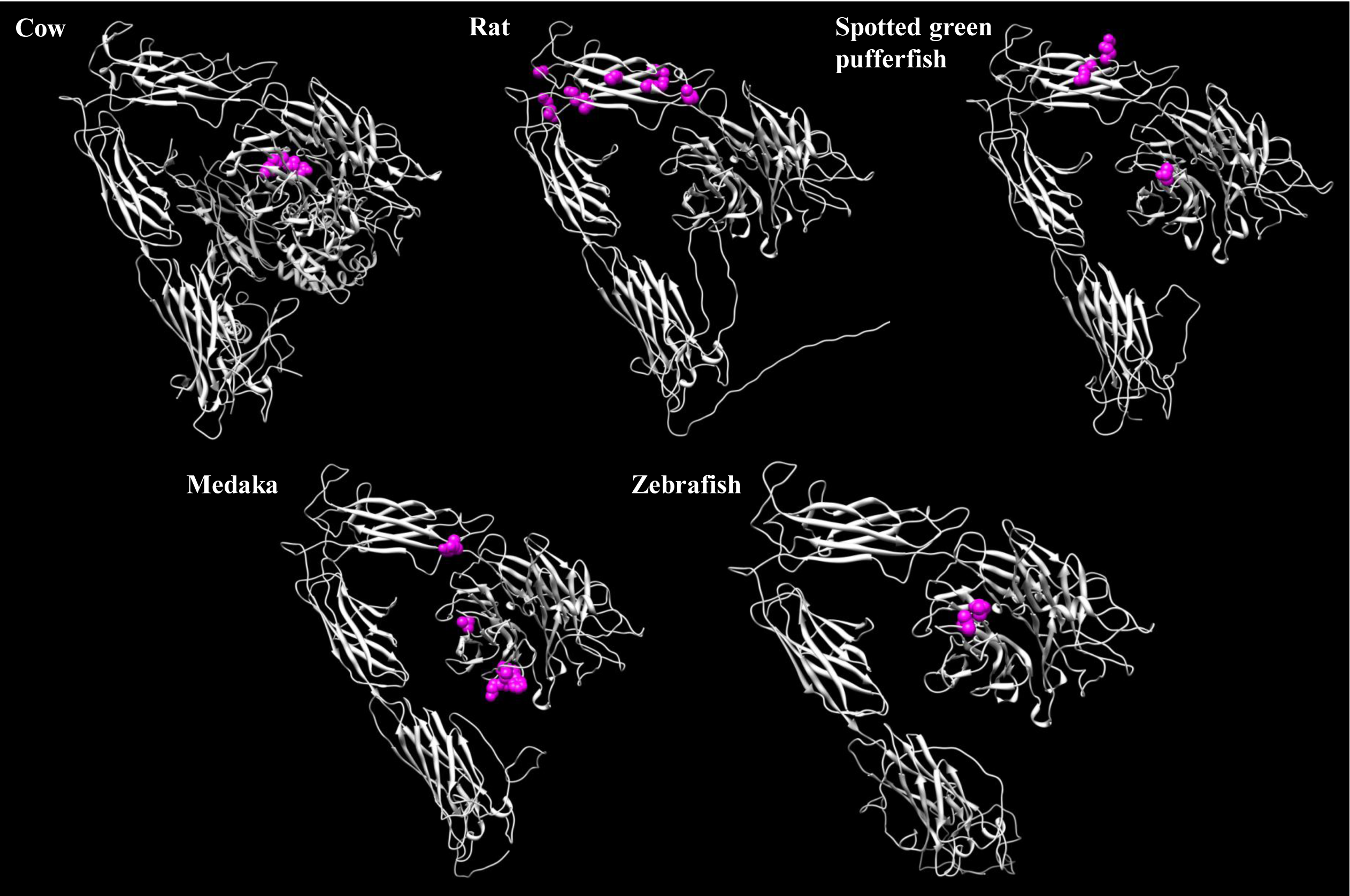

Supplement: Figure S3 — 3D structures of ITαv in cow, rat, green spotted pufferfish, medaka and zebrafish. Four structures were modeled with human αvβ3 integrin as template (PDB: 1L5G) [108]. The structure presented for the cow is the template itself. Amino acids under positive selection are in pink. This integrin is expressed by both oocyte and sperm. The pretreatment of sperm with anti ITαv antibodies significantly decreases the sperm-zona pellucida binding and the fertilization [109]. (TIF) [file pone.0044548.s003.tif]

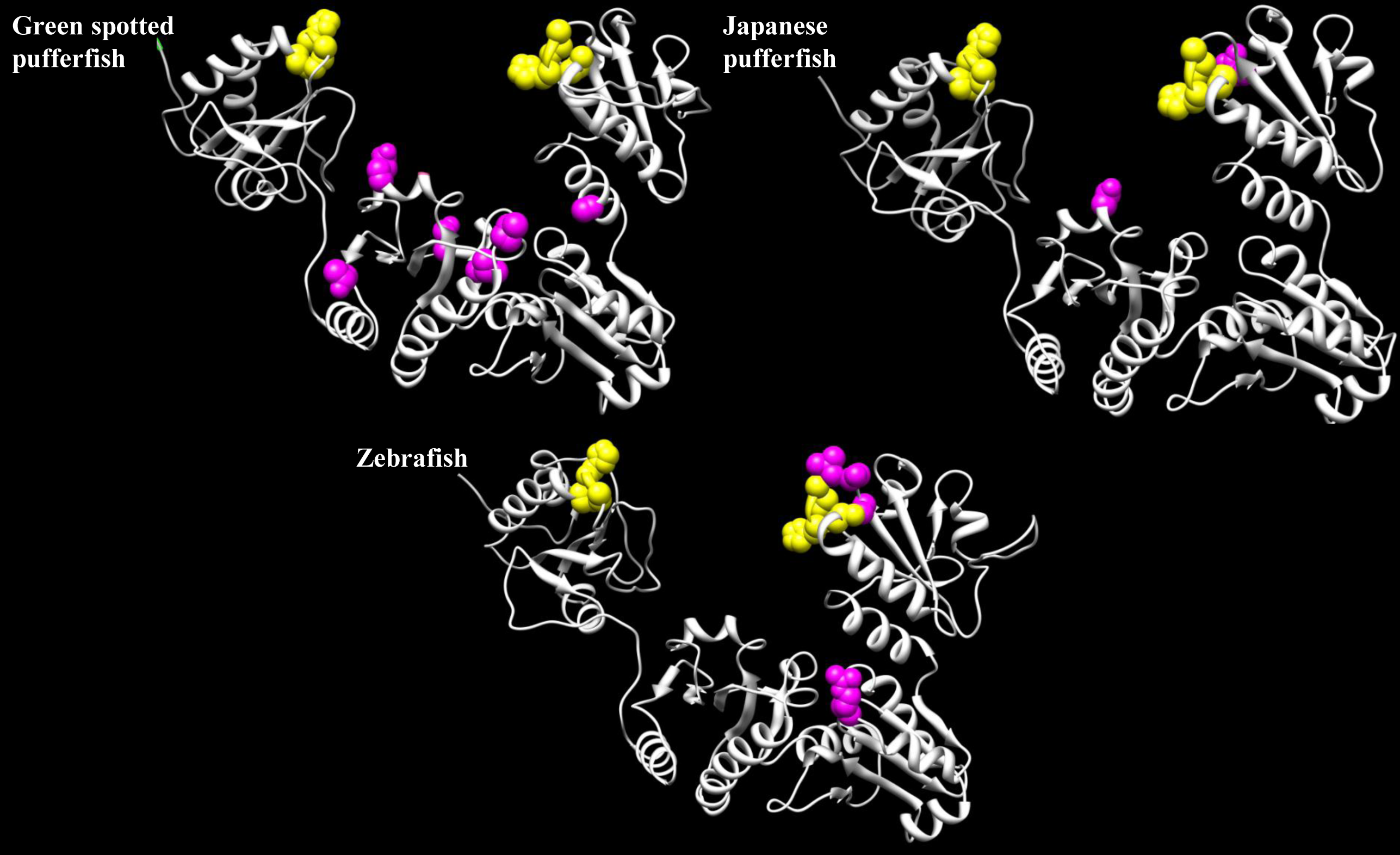

Supplement: Figure S4 — 3D structures of PDIA3 in green spotted pufferfish, japanese pufferfish and zebrafish. The three structures were modeled with human ERp57 as template (PDB: 3F8U) [110]. Catalytic motifs of the protein are in yellow, amino acids under positive selection are in pink. PDIA3 is expressed on sperm in the acrosomal region. Its role in the fusion process of sperm and oolemma was demonstrated with the use of anti-PDIA3 antibodies [89]. (TIF) [file pone.0044548.s004.tif]

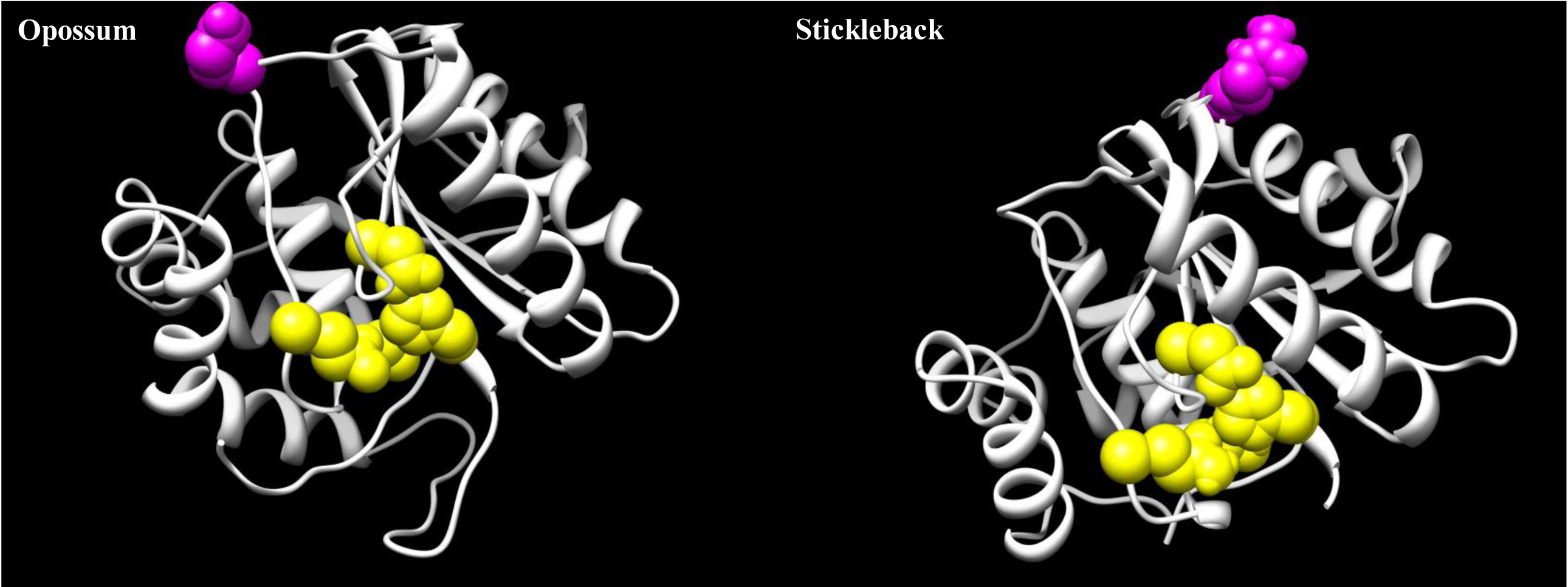

Supplement: Figure S5 — Models of the 3D structures of UCHL3 in opossum and stickleback. The two structures were modeled with human UCHL3 as template (PDB: 1UCH) [111]. Amino acids corresponding to the catalytic site of the enzyme are represented in yellow, amino acids under positive selection in pink. UCHL3 protein is present on both sperm acrosome and oocyte cortex. A study showed that UCHL3 is an ubiquitin C-terminal hydrolase and is involved in antipolyspermy defense during porcine fertilization [78]. (TIF) [file pone.0044548.s005.tif]

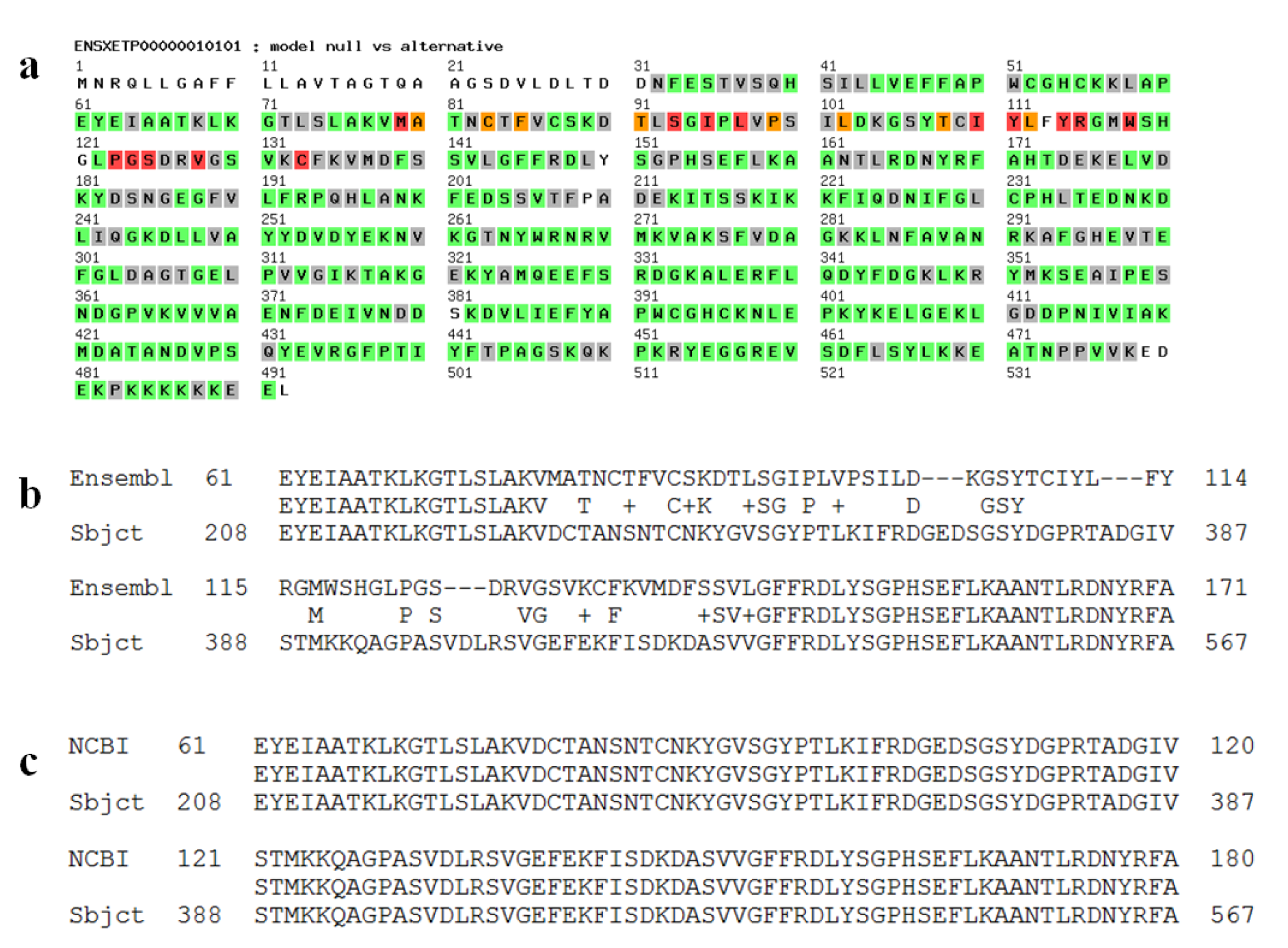

Supplement: Figure S6 — An example of a sequence error in Ensembl. The sequence presented in (a) is the protein sequence of the gene PDIA3 from Xenopus. Amino acids in red and orange are those which have been detected as positively selected by PhyleasProg. A tBLASTn has been performed with this sequence against EST database of NCBI. The result of the BLAST is presented in (b), the domain which contains amino acids under positive selection in (a) is not retrieved. A new sequence for Xenopus was searched in NCBI RefSeq database. The sequence of the best BLAST hit is submitted to a tBLASTn against the EST database of NCBI for verification (c). The new identified sequence from NCBI replaced in this case the sequence from Ensembl. (TIF) [file pone.0044548.s006.tif]

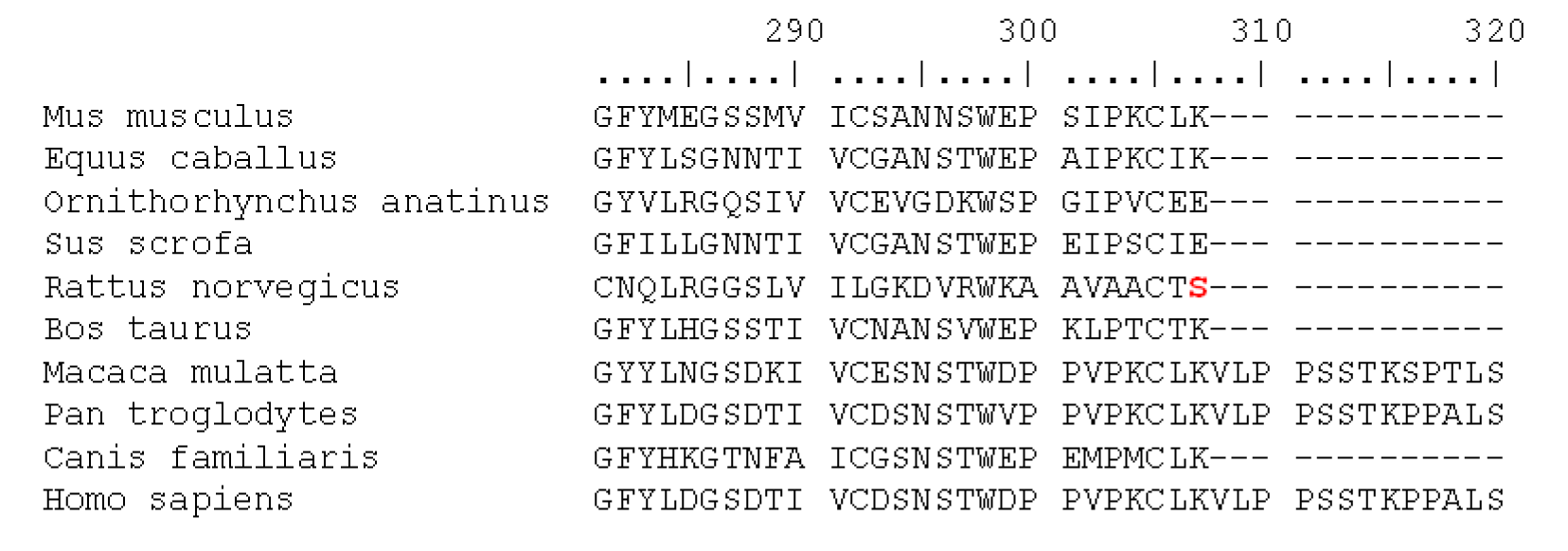

Supplement: Figure S7 — An example of a doubtful amino acid under positive selection. The amino acid in red in the sequence of the rat (Rattus norvegicus) was found as positively selected in the alignment of protein sequences of the gene CD46. Because it is located at the boundary of the alignment, this amino acid is not considered, because doubtful. (TIF) [file pone.0044548.s007.tif]

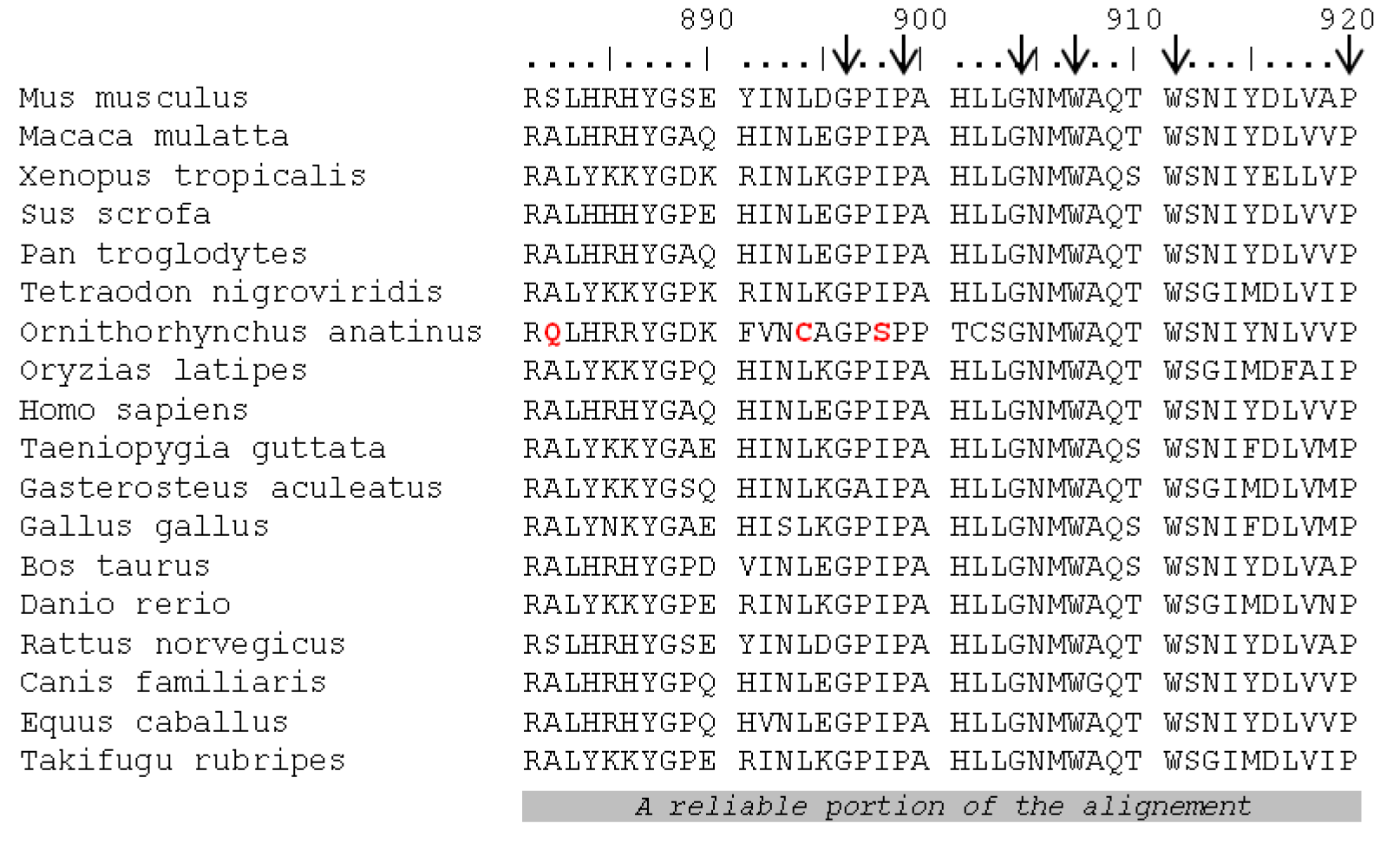

Supplement: Figure S8 — An example of reliable amino acids under positive selection. Amino acids indicated in red in the sequence of the platypus (Ornithorhynchus anatinus) are considered as reliable in this multiple sequence alignment of ACE protein sequences. (TIF) [file pone.0044548.s008.tif]

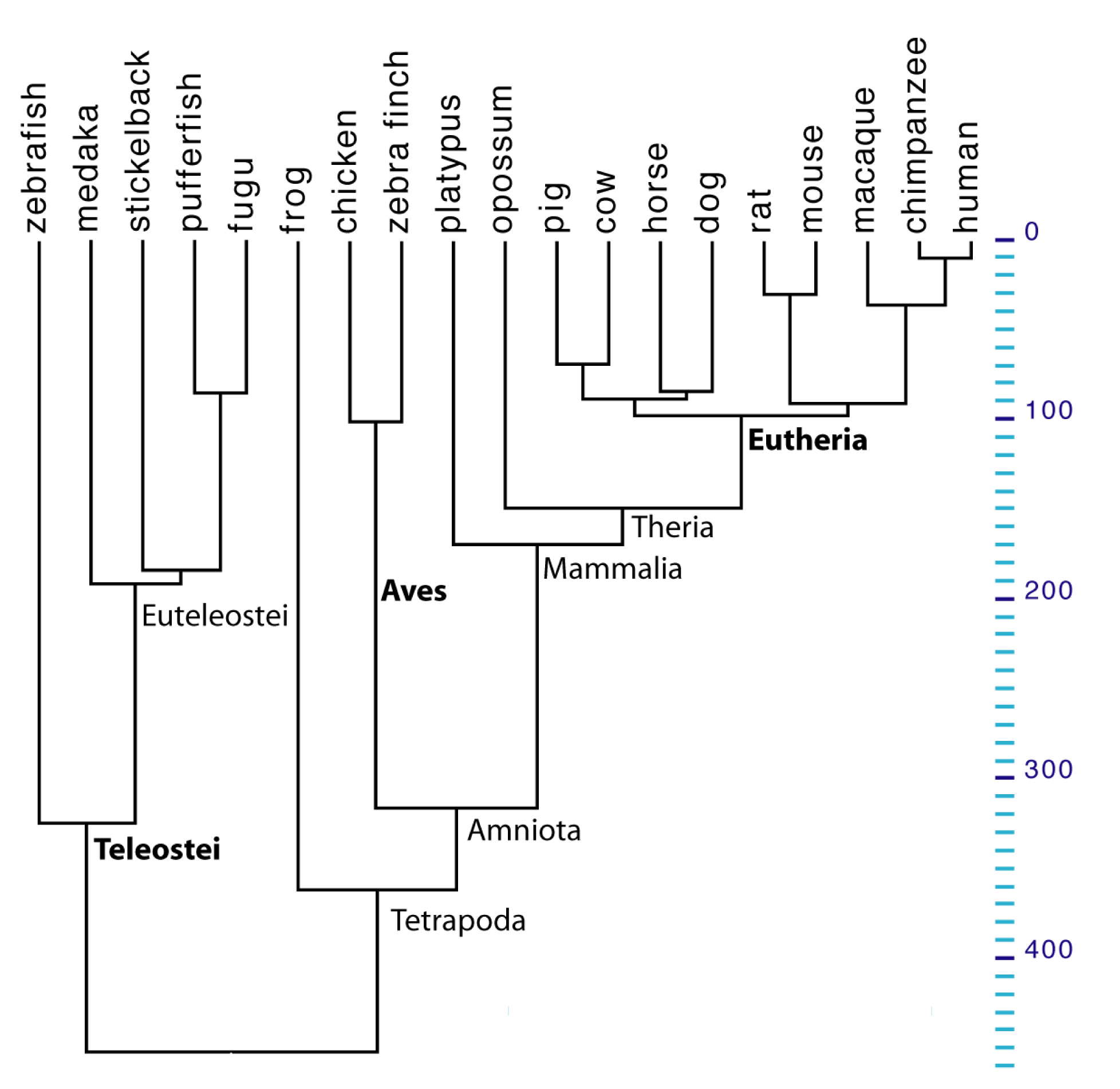

Supplement: Figure S9 — Time-calibrated reference phylogeny used for all phylogeny-informed analyses. This tree follows classical time-calibrated phylogenies of teleosts [112] and mammals [113]. The divergence time between the chicken and zebra finch follows a recent review paper [114]. The other divergence times derive from a review of paleontological and molecular dates [115]. The names of several higher taxa are placed, with those most discussed in the text in bold type. The scale to the right gives approximate divergence times (in millions of years). (TIF) [file pone.0044548.s009.tif]
