# Supplementary material for: Evolution of Genes Involved in Gamete Interaction: Evidence for Positive Selection, Duplications and Losses in Vertebrates
Source: PLoS One. 2012 Sep 5;7(9):e44548. doi: 10.1371/journal.pone.0044548 (PMC3434135; doi:10.1371/journal.pone.0044548)
Supplement: Table S1 — Genes included in the analysis. (DOCX) [file pone.0044548.s010.docx]

**Table S1. Genes included in the analysis**

| **Function** | **Gene** | **Gene Ensembl ID** | **Species** | **Full name of the gene** | **Reference** |
| --- | --- | --- | --- | --- | --- |
|  | CRISP1 | ENSP00000338276 | Human | cysteine-rich secretory protein 1 | [42] |
| Binding | ZPB/ZP4 | ENSP00000355529 | Human | zona pellucida glycoprotein 4 | [43] |
|  | ZPA/ZP2 | ENSMUSP00000033207 | Mouse | zona pellucida glycoprotein 2 | [44] |
|  | ZPAX | ENSGALP00000026516 | Chicken | similar to egg envelope component ZPAX | [45] |
|  | Ace | ENSMUSP00000001963 | Mouse | angiotensin I converting enzyme | [46] |
|  | Mfge8 | ENSMUSP00000032825 | Mouse | milk fat globule-EGF factor 8 protein | [47] |
|  | C1QBP | ENSBTAP00000013734 | Cow | Complement component 1 Q subcomponent-binding protein, mitochondrial Precursor | [48] |
|  | Bsg | ENSMUSP00000070751 | Mouse | basigin | [49] |
|  | SPA17 | ENSMUSP00000002013 | Rabbit | Sperm surface protein Sp17 | [50] |
|  | ACR | ENSP00000216139 | Human | Acrosin | [51] |
|  | Man2c1 | ENSRNOP00000044794 | Rat | Alpha-mannosidase 2C1 | [52] |
|  | Zpbp2 | ENSMUSP00000017339 | Mouse | zona pellucida binding protein 2 | [53] |
|  | B4GT1 | ENSBTAP00000020286 | Cow | Beta-1,4-galactosyltransferase 1 | [54] |
|  | Clgn | ENSMUSP00000002259 | Mouse | calmegin | [55] |
|  | DCXR | ENSP00000303356 | Human | dicarbonyl/L-xylulose reductase | [56] |
|  | Hspe1 | ENSMUSP00000074724 | Mouse | heat shock protein 1 (chaperonin 10) | [57] |
|  | ZPBP | ENSSSCP00000016566 | Pig | zona pellucida binding protein | [58] |
|  | ZPC/ZP3 | ENSP00000378326 | Human | zona pellucida glycoprotein 3 (sperm receptor) | [43] |
|  | ZAN | ENSSSCP00000008195 | Pig | zonadhesin | [59] |
|  | Adam1a | ENSMUSP00000098320 | Mouse | a disintegrin and metallopeptidase domain 1a | [60] |
|  | Adam1b | ENSMUSP00000078343 | Mouse | a disintegrin and metallopeptidase domain 1b | [60] |
|  | Adam3 | ENSMUSP00000033958 | Mouse | a disintegrin and metallopeptidase domain 3 (cyritestin) | [61] |
|  | Spermadhesins (AWN) | ENSSSCP00000003227 | Pig | Spermadhesin AWN | [62] |
|  | Cstf2t | ENSMUSP00000093831 | Mouse | cleavage stimulation factor, 3' pre-RNA subunit 2, tau | [63] |
|  | ZPD | ENSGALP00000001597 | Chicken | zona pellucida protein D | [64] |
|  | Adam2 | ENSMUSP00000022618 | Mouse | a disintegrin and metallopeptidase domain 2 | [65] |
|  | FUT5 | ENSP00000252675 | Human | fucosyltransferase 5 (alpha (1,3) fucosyltransferase) | [66] |
|  | Glipr1l1 | ENSMUSP00000073302 | Mouse | GLI pathogenesis-related 1 like 1 | [67] |
|  | PTGDS | ENSBTAP00000020065 | Cow | Prostaglandin-H2 D-isomerase Precursor | [68] |
|  | Spag8 | ENSP00000340982 | Human | sperm associated antigen 8 | [69] |
|  | Zp3r | ENSMUSP00000045443 | Mouse | zona pellucida 3 receptor | [70] |
| Acrosome reaction | ARSA | ENSP00000216124 | Human | arylsulfatase A | [71] |
|  | NAGLU | ENSMUSP00000001802 | mouse | N-acetylglucosaminidase | [72] |
|  | Vps39 | ENSMUSP00000099559 | Mouse | vacuolar protein sorting 39 | [73] |
|  | Pla2g10 | ENSMUSP00000023364 | Mouse | phospholipase A2, group X | [74] |
|  | CAPZA3 | ENSMUSP00000038562 | Mouse | capping protein (actin filament) muscle Z-line, alpha 3 | [75] |
|  | Cplx1 | ENSMUSP00000038502 | Mouse | complexin 1 | [76] |
|  | Fuca1 | ENSMUSP00000030434 | Mouse | fucosidase, alpha-L- 1, tissue | [77] |
|  | FUCA2 | ENSSSCP00000004465 | Pig | fucosidase, alpha-L- 2, plasma | [77] |
|  | Snap25 | ENSMUSP00000028727 | Mouse | synaptosomal-associated protein 25 | [73] |
|  | UCHL3 | ENSSSCP00000010107 | Pig | ubiquitin carboxyl-terminal esterase L3 (ubiquitin thiolesterase) | [78] |
|  | ZP1 | ENSP00000278853 | Human | zona pellucida glycoprotein 1 | [79] |
|  | Dkkl1 | ENSMUSP00000033057 | Mouse | dickkopf-like 1 | [80] |
|  | Prss21 | ENSMUSP00000024928 | Mouse | protease, serine, 21 | [81] |
|  | TEX101 | ENSP00000392573 | Human | testis expressed 101 | [82] |
| Fusion | FN1 | ENSBTAP00000010925 | Cow | Fibronectin Precursor | [83] |
|  | CD9 | ENSP00000371959 | Human | CD9 | [84] |
|  | Cd81 | ENSMUSP00000043768 | Mouse | CD81 antigen | [85] |
|  | ITGα6 | ENSMUSP00000107729 | Mouse | integrin, alpha 6 | [86] |
|  | Itgβ1 | ENSP00000364094 | Human | integrin, beta 1 | [87] |
|  | Itgβ3 | ENSMUSP00000021028 | Mouse | integrin beta 3 | [88] |
|  | ITGα5 | ENSBTAP00000018261 | Cow | Integrin alpha-5 Fragment | [83] |
|  | Pdia3 | ENSMUSP00000028683 | Mouse | protein disulfide isomerase associated 3 | [89] |
|  | CD46 | ENSP00000313875 | Human | CD46 molecule, complement regulatory protein | [90] |
|  | Spag9 | ENSRNOP00000003777 | Rat | sperm associated antigen 9 | [91] |
|  | ITα_V_ | ENSBTAP00000053614 | Cow | Integrin alpha-V Precursor (Vitronectin receptor subunit alpha)(CD51 antigen) | [92] |
|  | Igsf8 | ENSMUSP00000041232 | Mouse | immunoglobulin superfamily, member 8 | [93] |
|  | Itgα9 | ENSMUSP00000044227 | Mouse | integrin alpha 9 | [94] |
|  | CD151 | ENSP00000380565 | Human | CD151 molecule | [84] |
|  | CDH1 | ENSP00000261769 | Human | cadherin 1, type 1, E-cadherin | [95] |
|  | Uchl1 | ENSMUSP00000031131 | Mouse | ubiquitin carboxy-terminal hydrolase L1 | [96] |
|  | Adam15 | ENSMUSP00000029676 | Mouse | a disintegrin and metallopeptidase domain 15 (metargidin) | [97] |
|  | IZUMO1 | ENSP00000327786 | Human | izumo sperm-egg fusion 1 | [98] |
|  | Cdh2 | ENSRNOP00000021170 | Rat | Cadherin-2 Precursor, N-cadherin | [99] |
|  | Erp29 | ENSMUSP00000117347 | Mouse | endoplasmic reticulum protein 29 | [100] |
|  | Spaca3 | ENSMUSP00000069612 | Mouse | sperm acrosome associated 3 | [101] |
|  | CRISP2 | ENSMUSP00000024724 | Mouse | cysteine-rich secretory protein 2 | [102] |
|  | Spesp1 | ENSMUSP00000058522 | Mouse | sperm equatorial segment protein 1 | [103] |
|  | Spaca4 | ENSMUSP00000091991 | Mouse | sperm acrosome associated 4 | [104] |
